# Paratesticular Liposarcoma Presenting as an Inguinal Swelling: A Diagnostic Conundrum

**DOI:** 10.7759/cureus.62404

**Published:** 2024-06-14

**Authors:** Kausar A Fakih, Ashvi U Solanki, Sandhya Iyer, Santosh Menon, Riddhi Solanki

**Affiliations:** 1 General Surgery, Lokmanya Tilak Municipal Medical College & General Hospital, Mumbai, IND; 2 Pathology, Tata Memorial Hospital, Mumbai, IND; 3 Hospital Administration, Indian Institute of Public Health Gandhinagar, Gandhinagar, IND

**Keywords:** mdm2, radical orchidectomy, dedifferentiation, liposarcoma, paratesticular tumour

## Abstract

Paratesticular tumours are rare malignancies that are frequently misdiagnosed on presentation. We present a case of an elderly male with a six-month history of painless, progressively increasing left inguinal swelling. On preliminary examination and investigation, the swelling was misdiagnosed as a lymph nodal mass. Subsequently, a magnetic resonance imaging study detected a lesion that was not distinct from the spermatic cord. Biopsy testing of the said lesion was suggestive of poorly differentiated spindle cell neoplasm. The patient then underwent a high inguinal orchidectomy. Histopathological examination confirmed the diagnosis of a high-grade paratesticular dedifferentiated liposarcoma with rhabdomyoblastic differentiation. Due to the rarity of such tumours, the need for adjuvant chemotherapy and radiotherapy is debated.

## Introduction

Paratesticular tumours are defined as tumours originating within the scrotum but not from the testis. They arise from the epididymis (4%), spermatic cord (76%), and tunica vaginalis(20%) [1]. Most paratesticular tumours are seen in middle-aged and elderly men. Paratesticular tumours encompass less than 5% of all intrascrotal tumour masses [2]. Most are benign and fewer than one-third are malignant, usually sarcomas [3]. Liposarcoma is a malignant tumour arising from mesenchymal cells. Paratesticular liposarcoma is a rare condition with fewer than 200 cases reported in the literature [4].

## Case presentation

A 67-year-old male presented with chief complaints of a painless, slow-growing, swelling in the left groin region for six months. The patient had no complaints of fever, loss of weight or appetite, trauma, or any other swelling. On examination, the patient had a firm, non-tender swelling in the left inguinal region of size 6x5 cm. There were no changes in the overlying skin. It was palpated separately above the testis. However, the spermatic cord could not be well appreciated. No cough impulse was elicited. The right testis and cord were normal. Clinical examination was consistent with an inguinal mass separate from the testis, possibly a lymph nodal mass. Considering the age of the patient, it was suspected to be a lymphoma.

**Figure 1:**
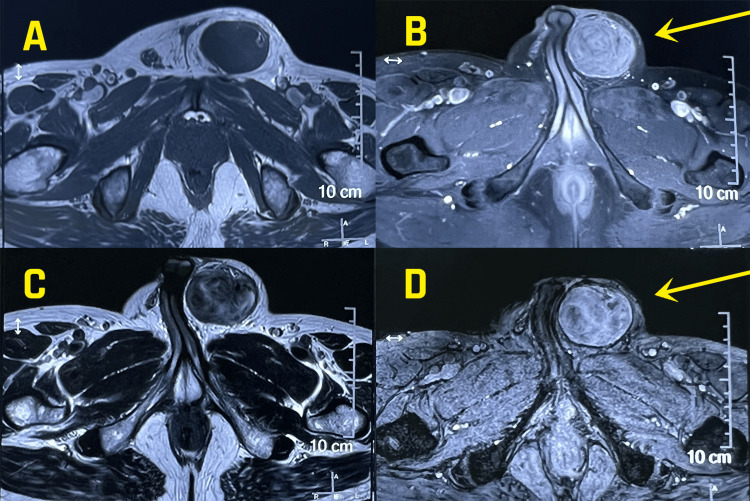
MRI axial view (A) and (B) showing T1W pre and post contrast images showing left inguinoscrotal mass with diffuse enhancement(arrow); (C) T2W image showing heterogenous signal in the lesion; (D) GRE image showing areas of blooming (arrow). GRE: gradient echo

**Figure 2:**
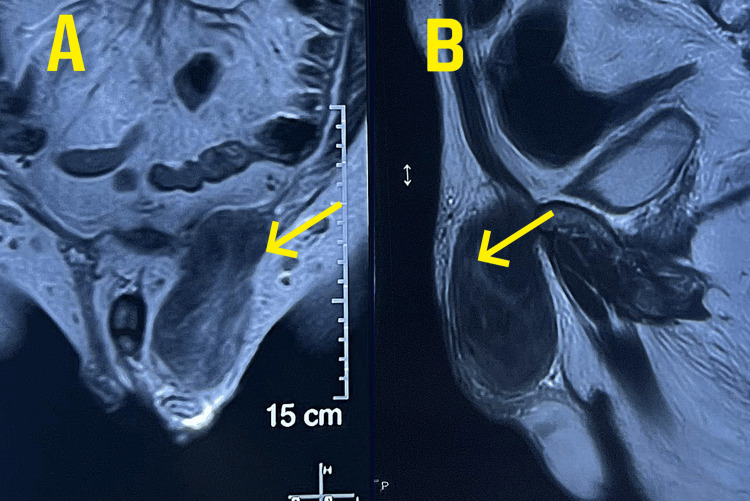
MRI (A) Coronal view (B) Sagittal view showing the left testicular mass seen separately from testis (as shown by arrows).

Tumour markers (beta human chorionic gonadotropin (hCG), alpha-fetoprotein (AFP), lactate dehydrogenase (LDH)) were within normal limits. Scrotal ultrasound was suggestive of a heterogeneous mass in the left inguinal region. For further characterisation of the lesion, the patient underwent an MRI pelvis (Figures 1, 2), which showed a 5x4x8 cm-sized T2 heterogenous mass lesion in the left inguinal region. The lesion showed significant post-contrast enhancement and areas of bleed within showing blooming on gradient echo (GRE) and restriction on DWI images. It was not seen separately from the left spermatic cord. Overlying scrotal wall oedema was seen. All these features were suggestive of a neoplastic aetiology.

A Tru-cut biopsy was done which was suggestive of a poorly differentiated tumour (likely spindle cell neoplasm). Immunohistochemistry (IHC) for the same was strongly positive for Ki67 and desmin. Computed tomography (CT) thorax and abdomen done for staging did not reveal any lymphadenopathy or distant metastasis.

**Figure 3:**
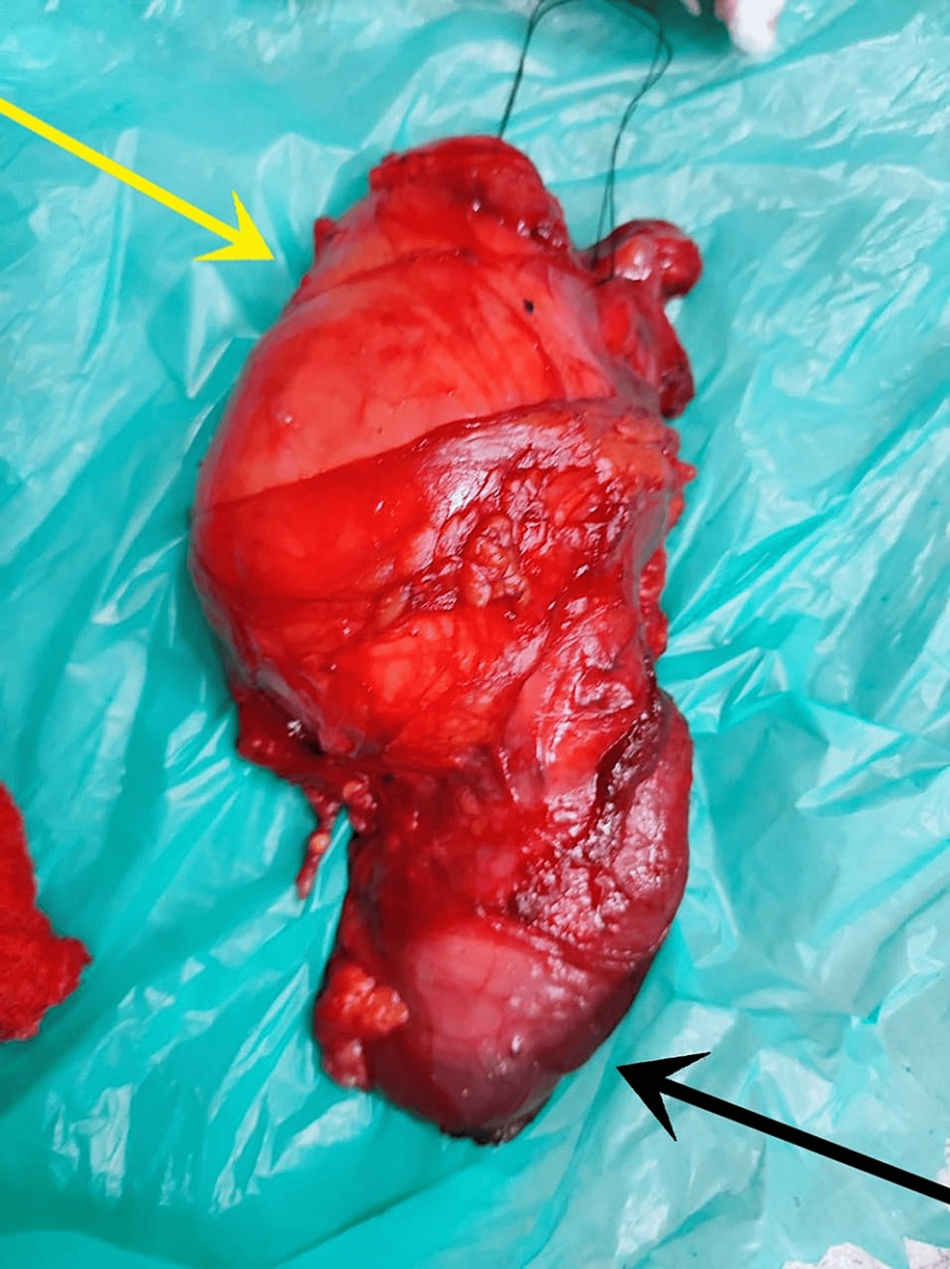
Gross image showing paratesticular mass (yellow arrow) with testis seen inferior to the mass (black arrow).

The patient was posted for left-sided high inguinal orchidectomy. Intraoperative findings included a 9x7x6 cm left-sided paratesticular tumour extending into the inguinal canal up to the deep ring (Figure 3). Wide local excision of the mass along with the left testis and inguinal canal contents up to the deep inguinal ring was done. The postoperative period was uneventful.

**Figure 4:**
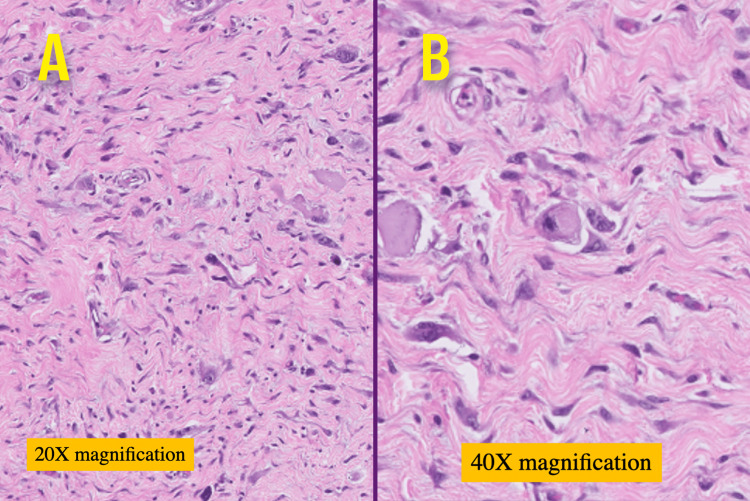
Microscopy hematoxylin and eosin stains (A) 20x (B) 40x magnification showing high-grade spindle cell sarcoma, exhibiting spindle cells arranged in interlacing fascicles along with singly scattered marked pleomorphic tumour cells in loose stroma with presence of lymphocytic follicles.

**Figure 5:**
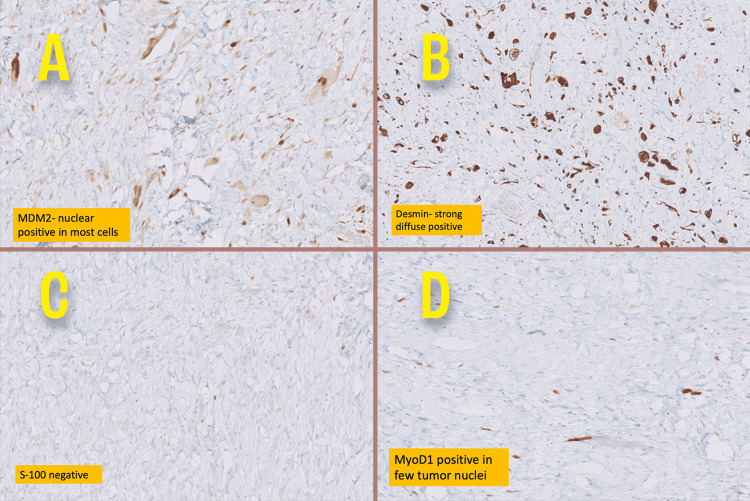
Immunohistochemistry stains in 20x magnification. (A) MDM2: nuclear positive in most cells; (B) Desmin: diffuse positive; (C) S-100: negative; (D) MyoD1: positive in few tumour nuclei.

Histopathologic examination (Figure 4) of the specimen confirmed a high-grade paratesticular dedifferentiated liposarcoma with rhabdomyoblastic differentiation (80%) with margins free of tumour (spermatic cord cut margins), and Fédération Nationale des Centres de Lutte Contre le Cancer (FNCLC) Grade 3 (3+3+0). IHC (Figure 5) of the specimen was positive for smooth muscle actin (SMA), desmin (diffused and strong), MyoP1 (focal and strong), murine double minute 2 (MDM2) (strong and nuclear) and negative for S100.

The patient was then advised to undergo radiotherapy due to the highly aggressive nature of the tumour, to reduce the risk of recurrence. The patient was followed up for one year without any recurrence.

## Discussion

According to the 2020 World Health Organization (WHO) classification of sarcoma [5], there are five histopathological subtypes of liposarcoma, namely, well-differentiated, dedifferentiated, myxoid, pleomorphic and myxoid pleomorphic, the most common being well-differentiated [5]. Well-differentiated tumours are low-grade tumours with a more indolent course.

Dedifferentiated liposarcomas (DDL) are aggressive tumours with a high risk of metastasis. DDL is a type of tumour that transitions from its initial well-differentiated liposarcomatous state to a non-liposarcomatous form. Dedifferentiation is a time-dependent process in 10-15% of well-differentiated forms, the average period being 7.7 years, and the five-year survival rate is 28% [1]. However, in our case, the patient had the swelling for only six months. The local recurrence rate of DDL is 40% and the metastatic rate is 15-30% [6].

The most common presentation of a paratesticular tumour is scrotal swelling. In our case, the patient had painless inguinal swelling for six months. Because of its extremely complex anatomical relationship, it is often misdiagnosed as inguinal hernia, lipoma, hydrocele, hematoma cavity, epididymitis, orchitis, lymph node or testicular tumour [7]. Clinically it is difficult to differentiate between benign and malignant tumours. The diagnosis of paratesticular tumours is documented by ultrasonography, CT, and MRI [8]. Sonography helps to differentiate paratesticular mass from a testicular mass. Features of malignancy seen on sonography are poorly defined disorganised solid masses with heterogeneity and hypervascularity. MRI is used to characterise the lesion and define its location. In our case, the MRI was suggestive of a mass in the inguinal canal with the possibility of neoplastic aetiology but no definitive diagnosis, so a Tru-cut biopsy was done. CT is the modality of choice to stage the tumour.

Due to the rarity of paratesticular liposarcoma, knowledge has been limited almost exclusively to case reports. In early tumours, the treatment of paratesticular sarcomas is surgery. A past study has shown that a significant number of patients undergoing simple excision had microscopic residual tumours [9]. Surgical resection should include a high radical inguinal orchidectomy with excision of surrounding tissue and ligation of the spermatic cord at the deep ring [9], which was also done for our patient, and tumour margins were free on histopathological examination. Incomplete excision is associated with frequent recurrence [8]. Khandekar et al. found that the three-year local recurrence-free survival was 100% for negative margins compared with 29% for positive margins [10]. Another such study by Kamitani et al. showed that three-year recurrence-free survival rates were significantly higher for those who underwent high inguinal orchidectomy than those who underwent tumorectomy (79.8 vs 54.1% respectively) [11]. Retroperitoneal lymph node dissection (RPLND) is indicated in the presence of suspicious lymphadenopathy seen on imaging, which was not present in our case [12]. The role of RPLND is debated.

The prognosis of paratesticular liposarcoma depends on the histological cell type. Currently, IHC of MDM2 and CDK4 may help screen for 12q13-15 amplification, which is seen in dedifferentiated tumours. In our case, MDM2 was strongly positive favouring the diagnosis of dedifferentiated liposarcoma [1]. Due to the rarity of cases, the role of adjuvant systemic chemotherapy in adults with paratesticular liposarcoma is not clear. Chemotherapy may be indicated in selected cases such as high-grade and metastatic tumours, agents that may be used are vincristine, cyclophosphamide, and doxorubicin. However, these tumours usually show chemoresistance, with response seen in less than one-third of patients, and no protocol has been established for the same [1].

Adjuvant radiotherapy can be considered for positive margin, unresectable and aggressive tumours [13]. Cerda et al. reported that adjuvant radiotherapy with a total dose of 54 Gy/27 or 30 fractions was found to have no recurrence in the median 18 months of follow-up (range 6-28 months) [13]. Postoperative follow-up should be at three, six, 12, and 24 months using CT scans, up to 10 years being mandatory [1].

## Conclusions

A diagnosis of paratesticular tumour should be kept in mind in a case of painless scrotal as well as inguinal swelling in a middle-aged man, as seen in our case. Surgery being the primary modality of treatment, radical orchidectomy with free surgical margins is recommended. The role of adjuvant radiotherapy and chemotherapy is controversial and needs to be studied further. Radiotherapy can be considered for dedifferentiated tumours due to their aggressive nature.
